# Optical coherence tomographic features of macular telangiectasia type 2: Korean Macular Telangiectasia Type 2 Study—Report No. 1

**DOI:** 10.1038/s41598-020-73803-9

**Published:** 2020-10-06

**Authors:** Young Ho Kim, Yoo-Ri Chung, Jaeryung Oh, Seong-Woo Kim, Christopher Seungkyu Lee, Cheolmin Yun, Boram Lee, So Min Ahn, Eun Young Choi, Sungmin Jang, Kihwang Lee

**Affiliations:** 1 Department of Ophthalmology, Ajou University School of Medicine, 164 World Cup-ro, Yeongtong-gu, Suwon, 16499 South Korea; 2 Department of Ophthalmology, Korea University College of Medicine, Seoul, South Korea; 3 Department of Ophthalmology, Yonsei University College of Medicine, Seoul, South Korea; 4 Institute of Vision Research, Yonsei University College of Medicine, Seoul, South Korea; 5 Institute of Human Barrier Research, Yonsei University College of Medicine, Seoul, South Korea; 6 Present Address: Department of Ophthalmology, Korea University Anam Hospital, Korea University College of Medicine, Seoul, South Korea; 7 Present Address: Retina Center, Saevit Eye Hospital, Goyang, South Korea

**Keywords:** Diseases, Signs and symptoms

## Abstract

We analysed the imaging findings of macular telangiectasia (MacTel) type 2 in Korea using spectral domain optical coherence tomography (SD-OCT) and investigated their relationship with visual acuity and clinical stages. A retrospective multicentre cross-sectional study was conducted in six tertiary hospitals in Korea and included 129 patients. We analysed all the SD-OCT images encompassing the macular area. Hyporeflective cavities (77.7%) were the most frequently detected abnormalities in SD-OCT. Disruption of the external limiting membrane, ellipsoid zone, and interdigitation zone were found in 67 (40.4%), 87 (52.4%), and 94 eyes (56.6%), respectively. Four eyes (2.4%) had lamellar macular hole, and five eyes (3.0%) full-thickness macular hole. Neovascularisation, either subretinal or intraretinal, was found in 14 eyes (8.4%). Eyes with outer retinal hyperreflective band disruption had lower visual acuity than those without them. The presented characteristic clinical features of OCT in MacTel type 2 can not only aid in differentiating this disease from others but are also helpful for better judgement of the disease stage in daily clinical practice. Inner retinal hyporeflective cavities without outer retinal abnormalities on SD-OCT, although classified as severity scale 3, could be considered a relatively early stage in the disease process in terms of vision.

## Introduction

Macular telangiectasia (MacTel) type 2 is an idiopathic bilateral disease, characterised by macular capillary network alterations and specific changes in the neurosensory retina^1^. Along with the development of various imaging modalities, clinical findings were better described in The MacTel Project, which started in 2005^1^. However, these findings included only a limited portion of Asians, and few studies were performed among the Korean population^2,3^.

Spectral domain optical coherence tomography (SD-OCT) has allowed for very-high-speed acquisition of images with high resolution. This improved resolution can now provide more precise images of the macula, and OCT has become a valuable method for diagnosing and studying MacTel type 2. The purpose of this multicenter study was to investigate the demographics of MacTel type 2 patients and its clinical characteristics using multimodal imaging after the introduction of SD-OCT in Korea. In this part of the Korean Macular Telangiectasia Type 2 Study, i.e., Report No. 1, we focused on the SD-OCT findings of MacTel type 2 in Korean patients, while the manifestations of MacTel type 2 using the fundus photograph (FP), fundus autofluorescence (FAF), and fluorescein angiography (FAG) will be discussed in another report, i.e., Report No. 2 [Kim et al. (2019) Demographic and multimodal imaging features of macular telangiectasia type 2: Korean Macular Telangiectasia Type 2 Study—Report No. 2, unpublished].

## Results

### Patients and demographic characteristics

In total, 166 eyes of 84 Korean patients with findings of MacTel type 2 were finally included for the analysis. The mean age at the initial visit was 66.2 ± 10.3 years, and the patients were predominantly female (63 of 84 patients, 75.0%). Twenty-six of 84 patients (31.0%) had a history of diabetes mellitus, and 32 of 84 patients (38.1%) had a history of hypertension. The mean logMAR visual acuity of the eyes was 0.282 ± 0.243, and 117 of 166 eyes (70.5%) showed 20/40 or better Snellen visual acuity.

### Spectral domain optical coherence tomography

SD-OCT images were available and gradable in all study eyes, and 11 eyes (6.6%) had no structural abnormalities in OCT images. The associations of detailed manifestations on SD-OCT in MacTel type 2 patients with visual acuity are presented in Table 1. An internal limiting membrane (ILM) drape was detected in 81 eyes (48.8%). It should be noted that 31 of 121 eyes (25.6%) with inner retinal hyporeflective cavities were accompanied by foveal floor flattening, and all eyes with a flattened foveal floor configuration had an ILM drape. No difference was observed in the mean visual acuity of the eyes with or without inner retinal cavity (*P* = 0.424), whereas the eyes with an outer retinal cavity, either above or below the external limiting membrane (ELM), had significantly lower visual acuity compared with those without an outer retinal cavity (all *P* < 0.05, Table 1).

**Table 1:** Macular telangiectasia type 2 clinical features of spectral domain optical coherence tomography.

Variable	Present^a^		Mean logMAR VA (SE)^b^		*P* value*
	N	%	Present	Absent	
SD-OCT (ungradable 0 eyes)	166	100			
Heidelberg OCT	104	62.7			
Topcon 3D OCT	44	26.5			
Zeiss OCT	18	10.8			
Normal	11	6.6			
Asymmetry^c^ (N = 19)	7	36.8	0.108 (0.029)	0.078 (0.023)	0.270
Increased IR reflectivity^c^ (N = 19)	3	15.8	0.090 (0.032)	0.090 (0.022)	0.998
**IR hyporeflective cavities**	121	72.9	0.271 (0.024)	0.312 (0.050)	0.424
Centre involved^d^	111	91.7	0.259 (0.025)	0.390 (0.067)	0.067
Flattening of foveal floor^e^ (N = 121)	31	25.6	0.265 (0.045)	0.271 (0.028)	0.899
Internal limiting membrane drape	81	48.8	0.313 (0.029)	0.250 (0.032)	0.087
**OR hyporeflective cavities (above ELM)**	56	33.7	0.368 (0.044)	0.240 (0.025)	0.010
Centre involved	40	71.4	0.400 (0.049)	0.320 (0.078)	0.352
**OR hyporeflective cavities (below ELM)**	52	31.3	0.385 (0.042)	0.236 (0.027)	0.002
Centre involved	50	96.2	0.392 (0.042)	0.111 (0.000)	< 0.001
Collapsing OR layers	45	27.1	0.473 (0.040)	0.207 (0.022)	< 0.001
Disorganization of IR layers	52	31.3	0.422 (0.045)	0.206 (0.023)	< 0.001
HF	96	57.8	0.335 (0.035)	0.210 (0.026)	0.003
Clustered HF at the foveola	23	13.9	0.131 (0.032)	0.307 (0.026)	0.173
Increased reflectivity of the ONL	88	53.0	0.328 (0.036)	0.228 (0.027)	0.022
Increased reflectivity of ELM	27	16.3	0.258 (0.046)	0.287 (0.028)	0.586
**ELM disruption**	67	40.4	0.426 (0.042)	0.185 (0.022)	< 0.001
Centre involved	58	86.6	0.457 (0.046)	0.224 (0.062)	0.002
**EZ disruption**	87	52.4	0.391 (0.035)	0.163 (0.024)	< 0.001
Centre involved	79	90.8	0.402 (0.037)	0.232 (0.070)	0.024
**IDZ disruption**	94	56.6	0.367 (0.033)	0.174 (0.026)	< 0.001
Centre involved	88	93.6	0.378 (0.035)	0.127 (0.036)	< 0.001
Lamellar macular hole	4	2.4	0.359 (0.191)	0.280 (0.024)	0.683
Full thickness macular hole	5	3.0	0.636 (0.147)	0.272 (0.024)	0.016
Subretinal NV or other evidence of NV	14	8.4	0.553 (0.086)	0.258 (0.023)	0.001

*ELM* external limiting membrane, *EZ* ellipsoid zone, *HF* hyperreflective foci, *IDZ* interdigitation zone, *IR* inner retinal, *NV* neovascularisation, *ONL* outer nuclear layer, *OR* outer retinal, *SD-OCT* spectral domain-optical coherence tomography, *SE* standard error, *VA* visual acuity.

*P values were by generalised linear models and the generalised estimation equation method comparing the presence and absence of each finding.

^a^Proportion of each finding was calculated among gradable eyes.

^b^Mean logMAR VA was estimated by generalised linear models and the generalised estimation equation method to account for correlation between eyes.

^c^Asymmetry and increased inner retinal reflectivity are only evaluated at the very early stage (MacTel severity score 0–2).

^d^Centre involvement was defined as the presence of cavities within the foveal floor without the inner retinal layers or less than about 300–600 μm from the foveolar centre.

^e^Flattening of foveal floor was only evaluated in patients with inner retinal hyporeflective cavities. Clustered hyperreflective foci was analysed in patients with only inner retinal cavity and MacTel severity score 0–2.

Among the included eyes, 51 eyes (31.3%) showed disorganisation of retinal inner layers, and 45 eyes (27.1%) had collapsing outer retinal layers. These two findings were thought to be the main cause of retinal thinning or atrophy in MacTel type 2, and those eyes with these findings had significantly lower visual acuity when compared with the eyes without them (all *P* < 0.001).

**Figure 1:**
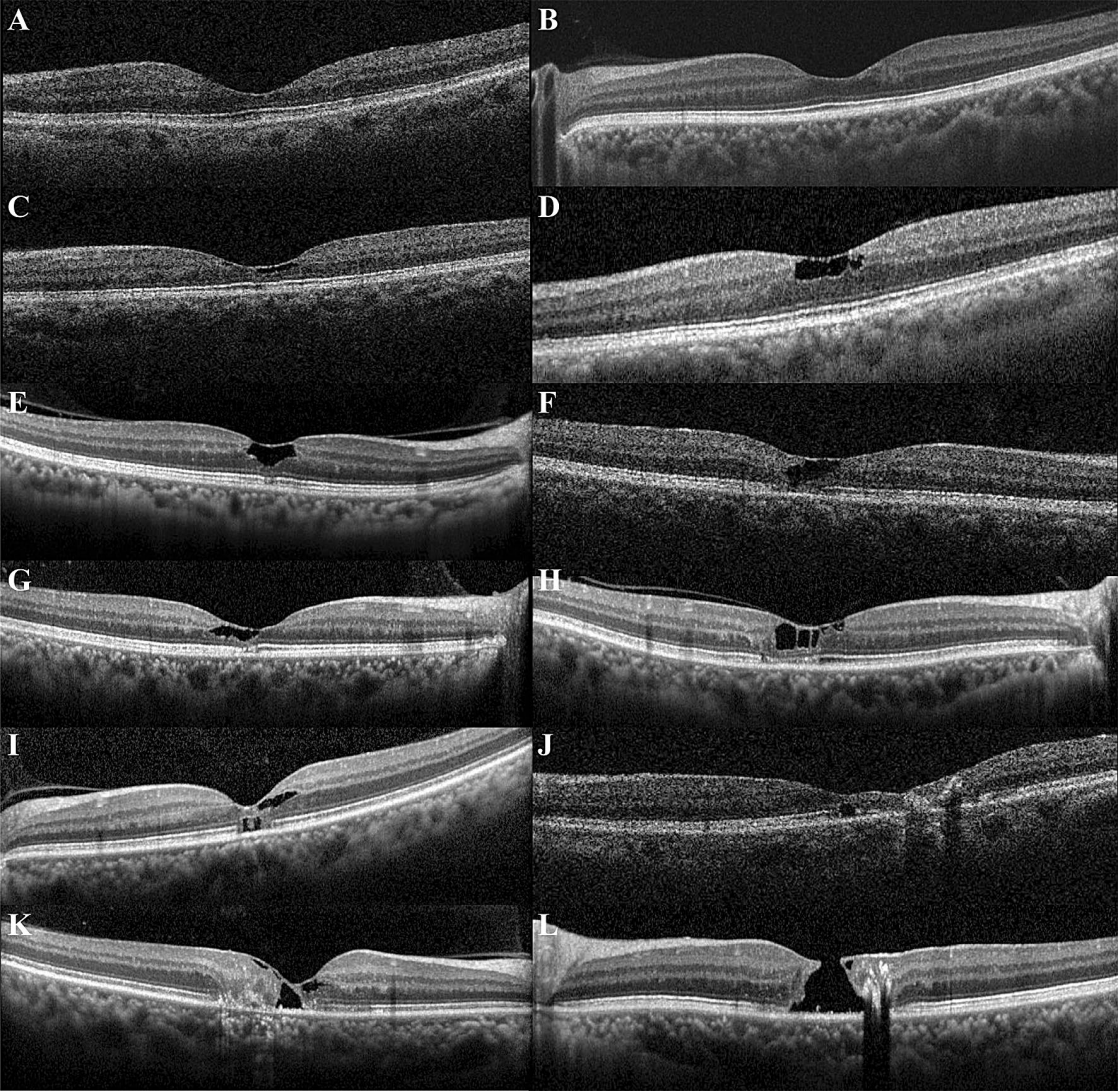
Various abnormalities found in OCT images of macular telangiectasia type 2 (MacTel type 2). (**A**) Early findings of MacTel type 2 in OCT images. Retinal thinning is present at the temporal parafovea, and an asymmetric foveal contour was found. (**B**) Increased reflectivity of the inner retina at the temporal parafovea and asymmetric foveal contour are present. (**C**) Inner retinal hyporeflective cavities and ILM drape are present. (**D**) There are several inner retinal hyporeflective cavities with tissue loss of the adjacent inner retinal layers and irregular boundaries. (**E**) An inner retinal hyporeflective cavity enlarged to more than the half of foveolar thickness but confined to the central subfield without adjacent inner retinal tissue loss. The ILM drape and foveal flattening are also visible. The ELM is well preserved, but focal disruption of the EZ and IDZ is present. (**F**) Inner retinal cavity can be found at the foveal centre and is continuous with the outer retinal cavity at the temporal parafovea. (**G**) Inner retinal cavity can be found at the foveal centre and is continuous with the outer retinal cavity at the foveal centre and temporal parafovea. (**H**) Inner retinal cavities are present at the foveola as well as the nasal and temporal parafovea. Outer retinal cavities at the foveolar and temporal parafovea were also accompanied by temporal parafoveal ELM defect and disruption of EZ and IDZ at the foveola and temporal parafovea. (**I**) Outer retinal cavity below the ELM and neighbouring focal disruption of EZ and IDZ of the foveola are present, whereas ELM is preserved with increased reflectivity above the outer retinal cavity. (**J**) Outer retinal cavity at the foveola is located below the ELM, which is accompanied by severe retinal thinning of the temporal parafovea. Concomitant intraretinal aggregated hyperreflective foci with posterior shadowing are present at the temporal parafovea. (**K**) Collapse of the outer retina and loss of inner retinal lamination are pronounced at the temporal parafovea. A large hyporeflective cavity of the foveola has been formed spanning from the inner retina to the outer retina both above and below the ELM. (**L**) A full-thickness macular hole is formed with tissue loss of the inner retina including ILM.

Disruption of the ELM, ellipsoid zone (EZ), and interdigitation zone (IDZ) were found in 67 (40.4%), 87 (52.4%), and 94 eyes (56.6%), respectively. As expected, the eyes with disrupted outer retinal hyperreflective bands had significantly lower visual acuity (all *P* < 0.001). Interestingly, six eyes had outer retinal cavities below the ELM at the foveal centre without both inner retinal cavities and outer retinal cavities above the ELM. These cases are difficult to diagnose as MacTel type 2 without FAG and FAF images, because typical MacTel findings such as collapsing of outer retinal layers and disorganisation of inner retinal layers are not found on OCT (Fig. 1).

Four eyes (2.4%) had a lamellar macular hole without an ILM drape, and five eyes (3.0%) had full-thickness macular hole. Neovascularisation, either subretinal or intraretinal, was found in 14 eyes (8.4%).

### Patterns of lateral extent of changes on SD-OCT

As mentioned in the methods, the centre on SD-OCT was defined as the foveal floor where there are no inner retinal layers. The most frequently found abnormalities involving the foveal centre are the inner retinal hyporeflective cavities (111 of 121 eyes, 91.7%), outer retinal hyporeflective cavities below the ELM (50 of 52 eyes, 96.2%), and disruption of the ELM (58 of 67 eyes, 86.6%), EZ (79 of 87 eyes, 90.8%), and IDZ (88 of 94 eyes, 93.6%), as presented in Table 2.

**Table 2:** Topographic extent of abnormalities of spectral domain optical coherence tomography.

Variables	Total (N = 166)	Foveal centre involved^a^					Foveal centre not involved		
		All	Only center	Temporal^b^	Nasal^b^	Temporal and nasal	All	Temporal	Nasal
IR hyporeflective cavities	121 (72.9)	111 (91.7)	42 (37.8)	25 (22.5)	6 (5.4)	38 (34.2)	10 (8.3)	4 (40.0)	6 (60.0)
OR hyporeflective cavities (above ELM)	56 (33.7)	40 (71.4)	14 (35.0)	16 (40.0)	0 (0)	10 (25.0)	15 (26.8)	13 (86.7)	2 (15.4)
OR hyporeflective cavities (below ELM)	52 (31.3)	50 (96.2)	37 (74.0)	9 (18.0)	0 (0)	4 (8.0)	2 (4.0)	2 (100)	0 (0)
Collapsing outer retinal layers	45 (27.1)	29 (64.4)	–	17 (58.6)	0 (0)	12 (41.4)	16 (35.6)	16 (100)	0 (0)
Disorganization of IR layers	52 (31.3)	31 (59.6)	–	24 (77.4)	0 (0)	7 (22.6)	21 (38.5)	21 (100)	0 (0)
ELM disruption	67 (40.4)	58 (86.6)	11 (19.0)	29 (50.0)	0 (0)	18 (31.0)	9 (13.4)	9 (100)	0 (0)
EZ disruption	87 (52.4)	79 (90.8)	27 (34.2)	34 (43.0)	0 (0)	18 (22.8)	8 (9.2)	8 (100)	0 (0)
IDZ disruption	94 (56.6)	88 (93.6)	34 (38.6)	35 (39.8)	0 (0)	19 (21.6)	6 (6.4)	6 (100)	0 (0)

Data are expressed as number (%).

*ELM* external limiting membrane, *EZ* ellipsoid zone, *IDZ* interdigitation zone, *IR* inner retinal, *OR* outer retinal.

^a^Foveal centre involvement was defined as abnormalities located within the foveal floor without the inner retinal layers or less than about 300–600 μm from the foveolar centre.

^b^The nasal side and the temporal side of fovea were classified depending on the relative position to the foveolar centre.

The location of the inner retinal hyporeflective cavities was limited within the lower half of foveal slope when presented only in the nasal fovea, whereas the temporal inner retinal cavities showed wider involvement of the lateral foveal slope. Most outer retinal hyporeflective cavities below the ELM presented at the foveal centre, and 74% involve the foveal centre without parafoveal involvement (Fig. 1).

Most eyes with collapsing outer retinal layers had disruption of the ELM (39 of 45 eyes, 86.7%), EZ (43 of 45 eyes, 95.6%), and IDZ (44 of 45 eyes, 97.8%) at the foveola. Eyes with disorganised retinal inner layers also had disruption of the ELM (43 of 52 eyes, 82.7%), EZ (49 of 52 eyes, 94.2%), and IDZ (50 of 52 eyes, 96.2%) at the foveola (see Supplementary Table S1).

### Subgroup analysis of MacTel severity 3

#### Hyporeflective inner retinal cavity with or without other OCT findings

More than two-thirds of the eyes examined (111 of 166 eyes, 66.9%) were classified into severity scale 3, which were further classified into two groups according to the OCT findings. The first group was classified as eyes that only have inner retinal hyporeflective cavities and the second group as the eyes that have other findings on OCT such as outer retinal hyporeflective cavities, collapsing outer retinal layers, and disorganised retinal inner layers with/without inner retinal hyporeflective cavities (Fig. 1).

The first group (50 eyes) had a significantly better visual acuity compared to the eyes with the other findings (61 eyes, *P* = 0.005) and had significantly less FP, confocal blue-light reflectance (CBR), FAF, early FAG, and late FAG scores (*P* = 0.005, 0.034, 0.012, 0.039, 0.010, and 0.003, respectively, Table 3). No eyes within the first group had disruption of the ELM at the foveal centre and nasal parafovea (see Supplementary Table S1).

**Table 3:** Subgroup analysis within MacTel severity 3.

Variable		Inner retinal cavity			Clustered HF at foveola		
		Only IR cavity	With other findings	*P* value*	Present	Absent	*P* value*
		(N = 50)	(N = 61)		(N = 19)	(N = 31)	
LogMAR VA	Mean (SE)^a^	0.187 (0.030)	0.308 (0.031)	0.005	0.106 (0.030)	0.229 (0.033)	0.006
	N^b^	50	61		19	31	
FP score^c^	Mean (SE)	1.06 (0.17)	1.61 (0.22)	0.034	0.68 (0.17)	1.07 (0.21)	0.154
	N	49	58		19	30	
CBR score^c^	Mean (SE)	2.55 (0.63)	3.67 (0.353)	0.012	1.25 (0.18)	3.50 (0.35)	< 0.001
	N	6	13		3	3	
FAF score^c^	Mean (SE)	1.61 (0.24)	2.27 (0.24)	0.039	1.06 (0.22)	1.74 (0.29)	0.062
	N	41	48		18	23	
Early FAG score^c^	Mean (SE)	1.63 (0.23)	2.34 (0.17)	0.010	1.43 (0.268)	1.83 (0.297)	0.322
	N	44	56		15	27	
Late FAG score^c^	Mean (SE)	1.85 (0.22)	2.67 (0.20)	0.003	1.44 (0.32)	1.98 (0.32)	0.198
	N	44	56		15	27	

*CBR* confocal blue light reflectance, *FAF* fundus autofluorescence, *FAG* fluorescein angiography, *FP* fundus photo, *HF* hyperreflective foci, *SE* standard error, *VA* visual acuity.

*P values are by generalized linear models and the generalized estimation equation method to account for correlation between eyes.

^a^Data are expressed as estimated marginal mean (standard errors). Each estimated mean was shown to account for correlation between eyes using generalized linear models and the generalized estimation equation method.

^b^The number of eyes that had gradable images for analysis. Some images are missing or ungradable for each image modality and expressed the number of eyes that included for analysis.

^c^The score was the sum of the affected ETDRS subfields of each image modality.

#### Clustered hyperreflective foci with only inner retinal hyporeflective cavity

**Figure 2:**
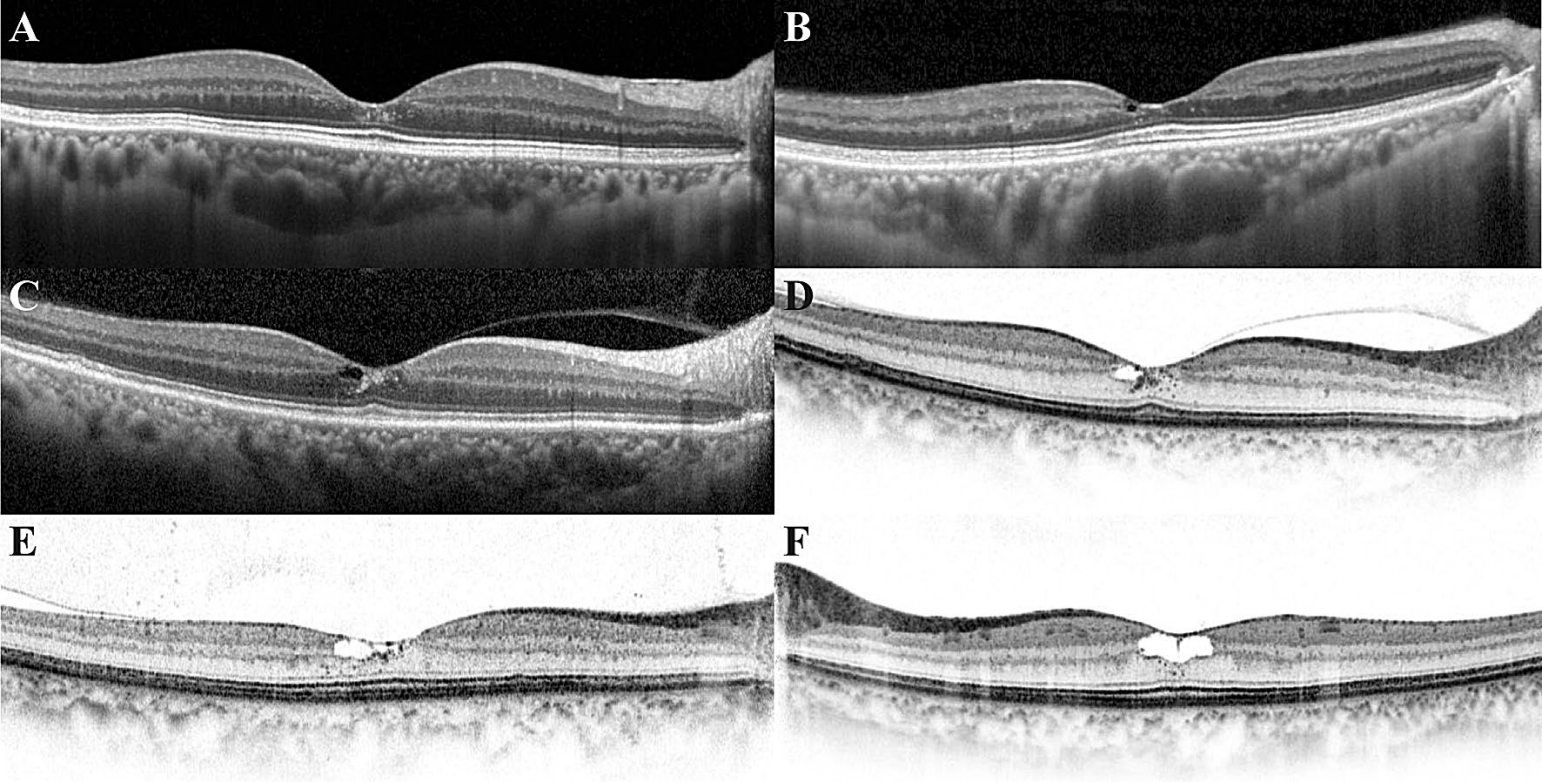
The presence of clustered hyperreflective foci (HF) at the foveola. HF are clustered where no retinal vessels are present within the foveal avascular zone. These HF are crowded around the border of the hyporeflective cavity or distributed vertically from the inner retina to the ELM. (**A**) Clustered HF can be found without a hyporeflective cavity. (**B**) A small inner retinal hyporeflective cavity is located within the lower half of the foveal slope, and HF are present just below the cavity. (**C**) Clustered HF are visible around the hyporeflective cavity, but the increased reflective foci appear like a retinal haze, which can be seen when retinal atrophy or fibrosis progresses. (**D**) The presence and path of clustered HF are more clearly visible at black on white mode. (**E**) Cluster HF present around the hyporeflective cavity with a dispersed pattern into the outer nuclear at temporal parafovea. (**F**) An inner retinal hyporeflective cavity enlarged to more than half of the foveolar thickness and clustered HF present at the border of the hyporeflective cavity.

To analyse the role of clustered hyperreflective foci (HF) at the foveola in the first group (Fig. 2), we further classified the first group into two categories: the eyes with or without clustered HF. Among 50 eyes with inner retinal hyporeflective cavities, there were 19 eyes (38%) with clustered HF and 31 eyes (62%) without HF (Table 3). Eyes with clustered HF had a significantly better visual acuity (*P* = 0.006) and lower CBR score (*P* < 0.001), whereas no differences were observed in the FP, autofluorescence, early FAG, and late FAG scores (*P* = 0.154, 0.062, 0.322, 0.198, respectively).

#### Eyes without late-phase hyperfluorescence in FAG image

**Figure 3:**
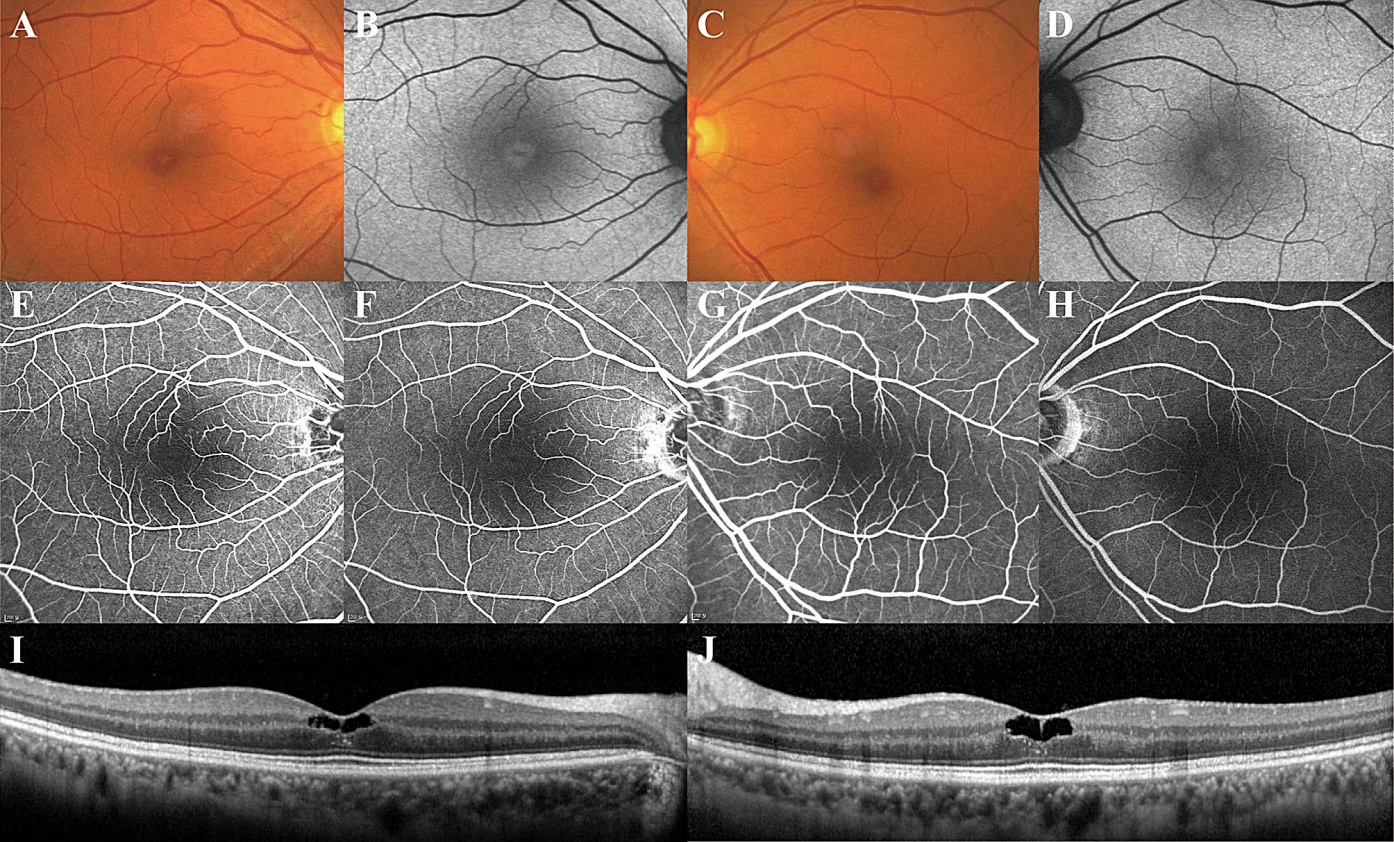
A case of macular telangiectasia type 2 without late-phase hyperfluorescence in FAG image. (**A**,**C**) In fundus photo, slight bilateral loss of retinal transparency and the features of a lamellar hole present temporally to the fovea. (**B**,**D**) In FAF image, increased autofluorescence is found at the foveal centre. (**E**–**H**) There were no definitive vascular abnormalities such as telangiectasia or dilated or tight-angled retinal vessels in early-phase FAG image. Remarkably, no vascular leakage or staining was noted at late-phase FAG images. (**I**,**J**) Inner retinal cavities with irregular boundaries and clustered hyperreflective foci around the cavity were noted on OCT.

A recent case report reported on a MacTel type 2 patient without clinically detectable vasculopathy^4^. We also found 8 of 111 eyes (7.2%) without late-phase hyperfluorescence (Fig. 3).

## Discussion

In this study, the characteristic findings on OCT imaging of Korean MacTel type 2 patients were summarised. The changes may present in various combinations and are most prominent at the foveal centre and temporal to the foveal centre. Among the various changes on OCT, abnormalities in outer retinal structures such as outer retinal hyperreflective band disruption, outer retinal hyporeflective cavities, and collapsing outer retinal layers were closely correlated with vision. Previously, Zhu et al.^5^ studied the relationship between the integrity of the ELM, inner retinal cavity, and photoreceptor disruption in MacTel type 2. They reported that the ELM was intact in all eyes with an inner retinal cavity without photoreceptor disruption. Consistent with previous results, the eyes with only inner retinal cavity showed no ELM disruption at the foveal centre in our subgroup analysis of patients with severity scale 3. The visual acuity of these eyes was significantly better than the eyes with other abnormal findings on OCT. Furthermore, these eyes displayed limited involvement of abnormal lesions in FP, FAF, and FAG images. Although these eyes were diagnosed as severity scale 3 due to atrophy on OCT images, it is thought that they are in the early phase of the disease in terms of vision.

It is interesting to note that there are many eyes with only the inner retinal cavity positioned vertically in the inner half of the foveola and horizontally located within the foveal slope. The morphological and locational features of this inner retinal cavity on OCT images are consistent with the histologically known location of the Müller cell cone (MCC), and it is suggested that these cavities might be related to the MCC damage. The MCC is known to have no contact to synapses or neuronal elements other than the central cones and lateral process that extend to the deep capillary plexus at the border between the inner nuclear layer and the outer plexiform layer^6,7^. This could be the reason for the findings of the only inner retinal cyst without ELM disruption. Furthermore, in our study, 25% of eyes with inner retinal hyporeflective cavities lost the foveal pit, showing a flattened foveal floor configuration accompanied by an ILM drape. Considering the MCC location and their plug-like function, which is important in maintaining the concavity of the fovea^6,8,9^, the development of the inner retinal hyporeflective cavity, foveal flattening, and the ILM drape are thought to be caused by MCC degeneration rather than the z-shape Müller cell in the parafoveal area that positioned across the whole retina. In some cases, the combined configuration of these abnormalities was similar to stages 0–1 of the macular hole on OCT image. However, the lamellar hole with ILM defect was very rare in MacTel type 2. Based on these findings, the pathogenesis of MacTel type 2 is different from the macular hole with an anteroposterior mechanical traction exerted on the MCC resulting in ILM disruption and cystoid space formation due to hydration^9^.

In the present study, the eyes with ELM, EZ, and IDZ disruptions, indicating photoreceptor damage, had worse visual acuity regardless of the foveal centre involvement. This is in accordance with the longitudinal study by Peto et al.^10^. They reported that the clinically meaningful loss of visual acuity occurred as the EZ disruption progresses to the foveal centre although a statistically significant modest loss of the mean vision was observed in the presence of EZ disruption on OCT^10^. In addition, a collapsed outer retinal layer and disorganised inner retinal layers had significantly associated with lower visual acuity. These features were frequently found in the temporal area and accompanied by foveolar ELM, EZ, and IDZ disruption. A typical Müller cell in the parafovea displays a z-shaped morphology and connects with the central cones in the foveola. These typical Müller cells form tight junctions with the inner segments of the photoreceptors that comprise the ELM^6,9,11^. In our study, ELM, EZ, and IDZ disruptions were more frequently observed in the eyes with an outer retinal cavity above the ELM at the foveolar, and these eyes had significant lower visual acuity than the eyes without it. As mentioned earlier, the outer process of the MCC vertically runs a straight course to the ELM, but the MCC does not form a functional column with photoreceptors and is not involved in supporting photoreceptor cell function^6^. Therefore, outer retinal abnormality findings might represent a more advanced stage of the disease when compared to the inner retina cavity.

We could commonly find clustered HF at the foveola in eyes with only an inner retinal hyporeflective cavity. HF could be related to various causes or signs in MacTel type 2^2,12–15^. Baumüller et al.^12^ suggested that HF might represent an early sign of a neurodegenerative process. In this study, 19 eyes with only inner retinal hyporeflective cavity had HF. Clustered HF were mainly found around the inner retinal cavity or vertically lined up from the inner retina to the ELM. The eyes with clustered HF had a significantly better visual acuity and lower CBR score than the eyes without clustered HF. It is noteworthy that four of the 19 eyes with HF had no late-phase hyperfluorescence in FAG image, which has a high correlation with vision. In addition, another four eyes in severity scale 0 or 2 had clustered HF without any retinal atrophy. These findings suggest that clustered HF may be associated with an earlier neurodegenerative process of MacTel type 2 than retinal cavitation as Baumüller et al.^12^ suggested. Its nature or origin is uncertain, but it could be one of the findings related to nonspecific neurodegeneration as proposed by Baumüller et al.^12^. Müller cell gliosis is characterised by both nonspecific response and specific response^16^. Our hypothesis is that the activated microglia is recruited to the injured retinal tissue or around the retinal cavity. Microglia has a key role in neuroinflammation as well as elimination of cell debris and deranged synapses^17^. Recently, HF on OCT image has been found in various retinal diseases such as diabetic retinopathy, age-related macular degeneration, and retinal vein occlusion, and some articles assume HF as microglia among many possibilities^18–21^. Further histopathologic or longitudinal study is required to determine the nature of clustered HF or the characteristics of its progression as the disease exacerbates.

This study had several limitations, including its observational retrospective nature and the fact that baseline images were analysed without longitudinal data. Further longitudinal and prospective studies can help describe the progressive and sequential nature of structural changes and the courses of the disease. Furthermore, the study images were acquired in routine clinical practice in multiple centres, and it was not possible to unify OCT equipment used in each centre. For the same reason, visual acuity was the only functional outcome assessed in this study even though the other functional measures such as reading acuity, scotoma size on microperimetry would be more conclusive.

In conclusion, the summarised characteristic clinical features of OCT in MacTel type 2 can not only aid in differentiating this disease from others, but are also helpful for better judgement of the disease stage in daily clinical practice. Inner retinal hyporeflective cavities without outer retinal abnormalities on SD-OCT, although classified as severity scale 3, could be considered as a relatively early stage in the disease process in terms of vision.

## Methods

This was a cross-sectional retrospective multicentre observational case series. We reviewed the medical records of 131 patients diagnosed with MacTel from the datasets of six hospitals from January 2009 to May 2019. The following hospitals participated in this study, and the Institutional Review Board (IRB) of each participating hospital approved this study, respectively, with each IRB numbers presented in Report No. 1: Ajou University Hospital (Suwon, Korea), Korea University Anam Hospital (Seoul, Korea), Korea University Guro Hospital (Seoul, Korea), Korea University Ansan Hospital (Ansan, Korea), Severance Hospital (Seoul, Korea), and Gangnam Severance Hospital (Seoul, Korea). This study complied with the tenets of the Declaration of Helsinki, and the informed consent was waived by the Institutional Review Board of each participating hospital given the retrospective nature of the study. Detailed methods of each imaging modalities except SD-OCT are described in Supplementary Methods.

BCVA, measured as the Snellen visual acuity ratio, was converted to a logMAR visual acuity for statistical analysis. The diagnosis of MacTel type 2 was made by the ophthalmologist of each patient, based on a constellation of signs of multimodal images using FP, FAG, and OCT images. The collected images were retrospectively analysed by a single retinal specialist (Y.H.K.), and multimodal images including FP, FAF, CBR, FAG, and OCT images were graded separately without additional clinical information.

### Spectral domain optical coherence tomography

SD-OCT of the macula was performed using 3D OCT-1000 Mark II, Cirrus HD-OCT (Models 4000 and 5000, Carl Zeiss Meditec, Dublin, CA, USA), SPECTRALIS HRA + OCT (Heidelberg Engineering, Heidelberg, Germany), and/or DRI OCT Triton (Topcon Corp., Tokyo, Japan). A horizontal line scan through the fovea and macular horizontal raster scan were acquired according to various protocols with a minimum 6 × 6 mm or 20° × 20° area and at least 49 B-scans per volume. All OCT images were averaged to improve the signal-to-noise ratio according to each device protocol. We analysed all the images encompassing the macular area and graded the findings as follows: asymmetry of the foveal depression; hyperreflectivity within the inner retinal layers; inner and outer retinal hyporeflective cavities; flattening of the foveal floor; ILM drape; loss of the outer nuclear layer or “collapse” of inner retinal layers onto the outer retina; disorganisation of retinal inner layers; increased reflectivity of the foveal centre, outer nuclear layer, and ELM; disruption of ELM, EZ, IDZ, and HF; lamellar macular hole or full-thickness macular hole; and subretinal neovascularisation or other evidences of neovascularisation.

Asymmetry of the foveal depression and hyperreflectivity within the inner retinal layers were evaluated in eyes presenting early signs of MacTel without hyporeflective cavities or other late findings. Asymmetry was defined when the thickness of the Early Treatment Diabetic Retinopathy Study (ETDRS) temporal inner ring was 10% thinner than the ETDRS nasal inner ring using the ETDRS thickness map.

Hyporeflective cavity was divided into the inner and outer cavities based on the border between the inner nuclear and outer plexiform layer. At the foveal floor where there were no inner retinal layers (about 300–600 μm at the foveolar centre), the cavity in contact with the inside surface or within half of the total thickness was expressed as the inner retina cavity. The outer retinal hyporeflective cavity was further divided into the upper ELM and lower ELM (Fig. 1).

We defined HF as discrete and well-circumscribed hyperreflective materials, sized 10–50 μm showing equal or higher reflectivity than the retinal pigment epithelium (RPE) band^18^. HF was evaluated at the foveolar avascular zone to exclude the effects of vascular leakage and under the ILM to exclude the HF resulting from a crystalline deposit.

### Grading of lateral extent of changes on OCT

In each horizontal line scan or raster scan, the foveal centre was defined as the floor of the fovea without inner retinal layers. This hyporeflective zone has a horizontal diameter about 300–600 μm wide at the foveolar centre. It should be noted that the inner retinal cavity presented within the lower third of the foveal slope with no inner retinal tissue loss of the adjacent outer foveal slope was graded with only the centre involved (Fig. 1). The nasal side and the temporal side of fovea were classified depending on the relative position to the foveolar centre.

### Severity scale

The severity scale definitions are as follows: severity 0, no evidence of disease (usually fellow eyes of affected individuals); severity 1, mild foveal AF changes without other abnormalities; severity 2, mild to moderate foveal hyperautofluorescence with angiographic abnormalities of MacTel; severity 3, moderate to marked foveal hyperautofluorescence with angiographic abnormalities and foveal atrophy documented on OCT; and severity 4, mixed patterns of fundus AF or marked thinning of the retina on OCT loss of photoreceptor and with accompanying RPE hyperplasia.

### Statistical analysis

All statistical analyses were performed using SPSS software version 25.0 (IBM Corp., Armonk, NY, USA). Descriptive statistical methods were used to delineate the basic characteristics of the participants, and the results were expressed as means (standard deviation/standard error) or proportion. Categorical variables were compared using the chi-square test or linear-by-linear association test and continuous variables using the independent t-test. Snellen visual acuity was converted to logMAR visual acuity for statistical analysis. To describe the association of various modality imaging findings of MacTel type 2 with visual acuity or other continuous variables, a generalised linear model with generalised estimation equations was used to account for the correlation between the eyes of the same patient. Results were considered statistically significant at *P* values < 0.05.

## Supplementary information

Supplementary Information.

Supplementary Table S1.
