# Supplementary material for: Optical coherence tomographic features of macular telangiectasia type 2: Korean Macular Telangiectasia Type 2 Study—Report No. 1
Source: Sci Rep. 2020 Oct 6;10:16594. doi: 10.1038/s41598-020-73803-9 (PMC7538897; doi:10.1038/s41598-020-73803-9)
Supplement: Supplementary file 1 — Supplementary Information. [file 41598_2020_73803_MOESM1_ESM.pdf]

Optical coherence tomographic features of macular telangiectasia type 2: Korean Macular Telangiectasia Type 2 Study – Report No. 1

Young Ho Kim[1,6*], Yoo-Ri Chung[1*], Jaeryung Oh[2], Seong-Woo Kim[2], Christopher Seungkyu Lee[3,4,5], Cheolmin Yun[2], Boram Lee[2], So Min Ahn[2], Eun Young Choi[3,4,5], Sungmin Jang[2,7], Kihwang Lee[1]

[1]Department of Ophthalmology, Ajou University School of Medicine, Suwon, Korea

[2]Department of Ophthalmology, Korea University College of Medicine, Seoul, Korea

[3]Department of Ophthalmology, [4]Institute of Vision Research, and [5]Institute of Human Barrier Research, Yonsei University College of Medicine, Seoul, Korea

[6]Present address: Department of Ophthalmology, Korea University Anam Hospital, Korea University College of Medicine, Seoul, Korea

[7]Present address: Retina Center, Saevit Eye Hospital, Goyang, Korea

[*]These authors equally contributed to this work.

Supplementary Methods

Color fundus photography, fundus autofluorescence image, confocal blue reflectance image

Various colour fundus photographs (FP) devices were used including AFC-210 (Nidek, Aichi, Japan), FF450 plus IR (Carl Zeiss Meditec AG, Jena, Germany), 3D OCT-1000 Mark II (Topcon Corp., Tokyo, Japan), and/or Optomap P200Tx/P200DTx (Optos, Dunfermline, Scotland). Preferably, 30° to 50° colour FP were used for analysis. However, ultrawide-field FPs were used in cases when there was no image below 50°. Analysis with stereoscopic FP could not be performed due to its retrospective nature.

Fundus autofluorescence (FAF) images were acquired using an Heidelberg Retinal Angiograph (HRA)/HRA 2, Spectralis HRA+OCT (Heidelberg Engineering, Heidelberg, Germany), and/or Optomap P200Tx/P200DTx. Confocal blue reflectance (CBR) images were acquired using HRA/HRA 2 or Spectralis HRA+OCT.

Gradings were based on methods published previously.[1-4] Colour FPs were graded for the presence or absence of classic features of MacTel type 2; loss of transparency (retinal graying), dilated and blunted retinal vessels, right-angled vessels, crystalline deposits, irregular retinal pigment epithelial abnormalities, pigment plaques or clumping, and/or evidence of neovascularisation. When the FP quality was low, FAF images were checked for any vascular abnormalities such as blunted and right-angled vessels. FAF images were graded for the presence of increased or decreased autofluorescence (AF).[1,5,6] Increased AF was defined as the presence of a higher greyscale value clearly detectable to the human eye. Decreased AF was categorised as the large area of decreased AF (larger than approximately 500 μm in diameter) or the localised decreased AF located mostly at the end of retinal vessels.[1,6] CBR images were graded for the presence of increased reflectance.

Fluorescein angiography

Fluorescein angiography (FAG) was performed with FF450 plus IR, HRA, HRA 2, Spectralis HRA+OCT, and/or Optomap P200Tx/P200DTx. Early-phase angiographic abnormality and late-

phase hyperfluorescence were analysed with the best image quality within 1 min and over 5 min, respectively. Early-phase images were graded for telangiectatic vessels, dilated retinal vessels, blunted retinal vessels, and right-angled vessels. Late-phase angiography images were graded for the lateral extent and depth of hyperfluorescence. Blocked fluorescence and choroidal neovascularisation were graded with early- and late-phase images of FAG.

References cited in Supplementary Methods

1 Clemons, T. E. *et al.* Baseline characteristics of participants in the natural history study of macular telangiectasia (MacTel) MacTel Project Report No. 2. *Ophthalmic Epidemiol* **17**, 66-73 (2010).

2 Charbel Issa, P. *et al.* Macular telangiectasia type 2. *Prog Retin Eye Res* **34**, 49-77 (2013).

3 Gass, J. D. & Blodi, B. A. Idiopathic juxtafoveolar retinal telangiectasis. Update of classification and follow-up study. *Ophthalmology* **100**, 1536-1546 (1993).

4 Wong, W. T. *et al.* Fundus autofluorescence in type 2 idiopathic macular telangiectasia: correlation with optical coherence tomography and microperimetry. *Am J Ophthalmol* **148**, 573-583 (2009).

5 Peto, T. *et al.* Correlation of clinical and structural progression with visual acuity loss in macular telangiectasia type 2: MacTel Project Report No. 6-The MacTel Research Group. *Retina* **38 Suppl 1**, S8-S13 (2018).

6 Balaskas, K. *et al.* Associations between autofluorescence abnormalities and visual acuity in idiopathic macular telangiectasia type 2: MacTel project report number 5. *Retina* **34**, 1630-1636 (2014).
