# Supplementary material for: Optical coherence tomographic features of macular telangiectasia type 2: Korean Macular Telangiectasia Type 2 Study—Report No. 1
Source: Sci Rep. 2020 Oct 6;10:16594. doi: 10.1038/s41598-020-73803-9 (PMC7538897; doi:10.1038/s41598-020-73803-9)
Supplement: Supplementary file 2 — Supplementary Table S1. [file 41598_2020_73803_MOESM2_ESM.pdf]

Optical coherence tomographic features of macular telangiectasia type 2: Korean Macular Telangiectasia Type 2 Study – Report No. 1

Young Ho Kim[1,6*], Yoo-Ri Chung[1*], Jaeryung Oh[2], Seong-Woo Kim[2], Christopher Seungkyu Lee[3,4,5], Cheolmin Yun[2], Boram Lee[2], So Min Ahn[2], Eun Young Choi[3,4,5], Sungmin Jang[2,7], Kihwang Lee[1]

[1]Department of Ophthalmology, Ajou University School of Medicine, Suwon, Korea

[2]Department of Ophthalmology, Korea University College of Medicine, Seoul, Korea

[3]Department of Ophthalmology, [4]Institute of Vision Research, and [5]Institute of Human Barrier Research, Yonsei University College of Medicine, Seoul, Korea

[6]Present address: Department of Ophthalmology, Korea University Anam Hospital, Korea University College of Medicine, Seoul, Korea

[7]Present address: Retina Center, Saevit Eye Hospital, Goyang, Korea

[*]These authors equally contributed to this work.

Supplementary Table S1. Lateral extent of disruption of outer retinal hyperreflective bands according to each representative OCT findings

Disruption	Lateral Extent	Inner retinal cavity				Collapsing outer retinal layers				Disorganization of retina inner layers			
		Only IR cavity (N=50)		With other findings (N=61)		Present (N=45)		Absent (N=121)		Present (N=52)		Absent (N=114)	
		N	%	N	%	N	%	N	%	N	%	N	%
ELM	Foveal center*	0	0.0	27	44.3	39	86.7	19	15.7	43	82.7	15	13.2
	Temporal fovea†	3	6.0	23	37.7	42	93.3	14	11.6	44	84.6	12	10.5
	Nasal fovea†	0	0.0	4	6.6	15	33.3	3	2.5	16	30.8	2	1.8
EZ	Foveal center*	7	14.0	41	67.2	43	95.6	36	29.8	49	94.2	30	26.3
	Temporal fovea†	3	6.0	26	42.6	42	93.3	18	14.9	44	84.6	16	14.0
	Nasal fovea†	0	0.0	3	4.9	14	31.1	4	3.3	16	30.8	2	1.8
IDZ	Foveal center*	11	22.0	45	73.8	44	97.8	44	36.4	50	96.2	38	33.3
	Temporal fovea†	4	8.0	26	42.6	42	93.3	18	14.9	44	84.6	16	14.0
	Nasal fovea†	0	0.0	4	6.6	15	33.3	4	3.3	16	30.8	3	2.6

ELM, external limiting membrane; EZ, ellipsoid zone; IDZ, interdigitation zone; IR inner retinal.

Foveal center was defined as abnormalities located within foveal floor without inner retinal layers or less than about 300-600 μm from foveolar center.

†The nasal side and the temporal side of fovea were classified depending on the relative position to the foveolar center.
